# Selective inhibition of histamine-evoked Ca^2+^ signals by compartmentalized cAMP in human bronchial airway smooth muscle cells

**DOI:** 10.1016/j.ceca.2017.12.002

**Published:** 2018-05

**Authors:** Philippa Dale, Victoria Head, Mark R. Dowling, Colin W. Taylor

**Affiliations:** a Department of Pharmacology,Tennis Court Road, Cambridge, CB2 1PD, UK; b Novartis Institutes for BioMedical Research, Fabrikstrasse, CH-4056, Basel, Switzerland; c Novartis Institutes for BioMedical Research Inc., 250 Massachusetts Avenue, Cambridge, MA, 02139, USA

**Keywords:** AC, adenylyl cyclase, 2-APB, 2-aminoethoxyphenylborane, ASM, airway smooth muscle, cAMP, 3',5'-cyclic AMP, COPD, chronic obstructive pulmonary disease, [Ca^2+^]_i_, intracellular free Ca^2+^ concentration, DMSO, dimethyl sulfoxide, EC_50_ (IC_50_), half-maximally effective (inhibitory) concentration, Epac, exchange protein activated by cAMP, GPCR, G protein-coupled receptor, hBASMC, human bronchial airway smooth muscle cell, HBS, Hepes-buffered saline, HBSS, Hank’s balanced salt solution, IBMX, 3-isobutyl-1-methylxanthine, IP_3_, inositol 1,4,5-trisphosphate, IP_3_R, IP_3_ receptor, LPA, 18:1 lysophosphatidic acid, pEC_50_, -logEC_50_, PKA, cyclic AMP-dependent protein kinase, PGE_2_, prostaglandin E_2_, PKI-myr, myristoylated PKA inhibitor, PLCβ, phospholipase C β, PTX, pertussis toxin, Airway smooth muscle, Ca^2+^ signaling, Cyclic AMP, Histamine, Protein kinase A, Spatial organization

## Abstract

Intracellular Ca^2+^ and cAMP typically cause opposing effects on airway smooth muscle contraction. Receptors that stimulate these pathways are therapeutic targets in asthma and chronic obstructive pulmonary disease. However, the interactions between different G protein-coupled receptors (GPCRs) that evoke cAMP and Ca^2+^ signals in human bronchial airway smooth muscle cells (hBASMCs) are poorly understood. We measured Ca^2+^ signals in cultures of fluo-4-loaded hBASMCs alongside measurements of intracellular cAMP using mass spectrometry or [^3^H]-adenine labeling. Interactions between the signaling pathways were examined using selective ligands of GPCRs, and inhibitors of Ca^2+^ and cAMP signaling pathways. Histamine stimulated Ca^2+^ release through inositol 1,4,5-trisphosphate (IP_3_) receptors in hBASMCs. β_2_-adrenoceptors, through cAMP and protein kinase A (PKA), substantially inhibited histamine-evoked Ca^2+^ signals. Responses to other Ca^2+^-mobilizing stimuli were unaffected by cAMP (carbachol and bradykinin) or minimally affected (lysophosphatidic acid). Prostaglandin E_2_ (PGE_2_), through EP_2_ and EP_4_ receptors, stimulated formation of cAMP and inhibited histamine-evoked Ca^2+^ signals. There was no consistent relationship between the inhibition of Ca^2+^ signals and the amounts of intracellular cAMP produced by different stimuli. We conclude that β-adrenoceptors, EP_2_ and EP_4_ receptors, through cAMP and PKA, selectively inhibit Ca^2+^ signals evoked by histamine in hBASMCs, suggesting that PKA inhibits an early step in H_1_ receptor signaling. Local delivery of cAMP within hyperactive signaling junctions mediates the inhibition.

## Highlights

• β_2_-adrenoceptors, via cAMP and PKA, inhibit histamine-evoked Ca^2+^ signals in human bronchial airway smooth muscle cells.
• Responses to other Ca^2+^-mobilizing receptors are unaffected or minimally affected by cAMP.
• There is no consistent relationship between the amounts of cAMP produced by different stimuli and inhibition of histamine-evoked Ca^2+^ release.
• Local delivery of cAMP within hyperactive signaling junctions stimulates PKA.
• PKA inhibits an early step in the signaling pathway activated by H_1_ histamine receptors.

## Graphical abstract

**Figure:**
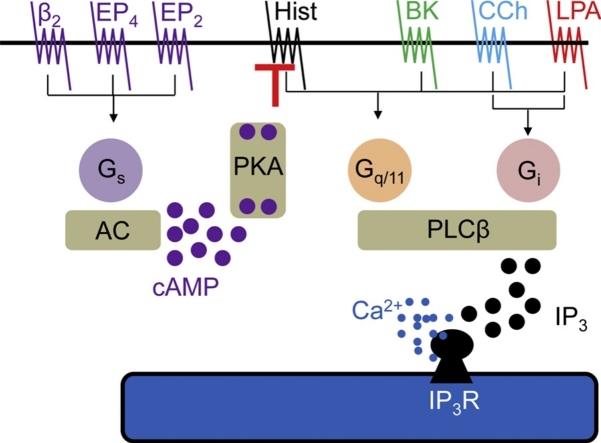

## 1 Introduction

Bronchial asthma and chronic obstructive pulmonary disease (COPD) are associated with inflammation, hyper-responsiveness and airway obstruction leading to restricted airflow. Although the nature of the inflammation and disease progression [[1], [2], [3]] differ for asthma and COPD, a major therapeutic target for both diseases is airway smooth muscle (ASM). Enhanced contractile activity and/or proliferation of ASM provoked by increased levels of acetylcholine, histamine, bradykinin or cytokines; by increased responsiveness to acetylcholine; or, after prolonged treatment with β-agonists, by attenuated activity of β_2_-adrenoceptors can all contribute to airway obstruction in asthma and COPD [[[1], [2], [3]],[[1], [2], [3]]]. Alongside anti-inflammatory therapies (e.g. inhaled glucocorticosteroids for asthma), management of both diseases relies heavily on inhaled drugs that induce relaxation of ASM via stimulation of β_2_-adrenoceptors (e.g. salbutamol or indacaterol) or antagonists of M_3_ muscarinic receptors (e.g. glycopyrronium bromide) to block contraction evoked by endogenous acetylcholine [[[1], [2], [3]],^4^]. Current therapies can provide some symptomatic relief for COPD, but they fail to prevent disease progression, and there are concerns about long-term use of β-agonists in asthmatic patients [5].

An increase in intracellular free Ca^2+^ concentration ([Ca^2+^]_i_) stimulates contraction of ASM, but additional mechanisms regulate the Ca^2+^-sensitivity of the contractile machinery, notably through RhoA and inhibition of myosin light chain (MLC) phosphatase [6]. Defects in Ca^2+^ signaling and the sensitization pathways are proposed to contribute to airway hyper-responsiveness [[7], [8], [9], [10]]. Ca^2+^ signals are usually initiated by receptors that stimulate phospholipase Cβ (PLCβ) and thereby formation of inositol 1,4,5-trisphosphate (IP_3_), which evokes Ca^2+^ release from the sarcoplasmic reticulum through IP_3_ receptors (IP_3_R). In human ASM, the major physiological contractile stimulus is acetylcholine released from parasympathetic terminals, which then stimulates PLCβ through M_3_ receptors and G_q/11_ [11], and possibly also through M_2_ receptors and G_i_ [12]. In diseased airways, contraction may be evoked by additional stimuli because the stimuli accumulate within the airways (e.g. bradykinin and histamine) [1] and/or their receptors are up-regulated (e.g. B_2_ bradykinin receptors) [13].

In ASM from various mammals, β-agonists cause relaxation and attenuate the increase in [Ca^2+^]_i_ evoked by receptors that stimulate PLC [5]. The mechanisms are not resolved, but there is evidence for reduced accumulation of IP_3_ [14], increased activity of the SR/ER Ca^2+^-ATPase (SERCA) [15], and inhibition of IP_3_Rs [16]. It has been widely supposed that cAMP and cAMP-dependent protein kinase (PKA) mediate these effects of β-agonists, but the evidence has been inconclusive [see discussion in 5] and there are suggestions that exchange proteins activated by cAMP (Epacs) may be more important than PKA [17,18]. Because ASM from different species respond to different stimuli [19] it is important to examine human cells, but there have been relatively few analyses of Ca^2+^ signaling in human ASM. The most informative studies have used either precision-cut lung slices, where the complex relationships between ASM and associated cells are maintained [19]; or cultured ASM, which bring the benefits of simplicity and availability, but with a risk that phenotypes may change in culture [20]. Hitherto, a major limitation of cultured human ASM has been loss of the muscarinic receptors [20,21] that both contribute to the contractile responses in COPD and asthma, and provide important targets for therapy.

Concern about long-term use of long-acting β-agonists has prompted interest in alternative therapies for asthma and COPD. These include prostaglandin E_2_ (PGE_2_), which can also stimulate adenylyl cyclase (AC), primarily through EP_2_ and EP_4_ receptors [22]. High concentrations of PGE_2_ are found in human lower respiratory tract [23] and they are increased further in eosinophilic bronchitis [24]. PGE_2_ relaxes human airways [22]; EP_2_ and EP_3_ receptors are upregulated in ASM from asthmatic patients [25]; and inhaled PGE_2_ may benefit patients with asthma or chronic bronchitis [22]. However, species differ in the responses of their ASM to PGE_2_ and in the EP receptors they express [22,26]. Even within human airways, there is conflicting evidence for the relative contributions of EP_2_ [27] and EP_4_ [22] receptors to relaxant responses. The effects of PGE_2_ on the Ca^2+^ signals evoked by contractile stimuli in human ASM are unknown. There is, therefore, a need in human ASM to establish whether PGE_2_ affects Ca^2+^ signals and through which receptors. Furthermore, there is evidence that β-agonists and PGE_2_ stimulate different isoforms of AC, thereby producing cAMP in different intracellular locations and with different functional consequences [see references in 28]. Hence, there is a need to determine in human ASM the interplay between the different G protein-coupled receptors (GPCRs) that stimulate Ca^2+^ and cAMP signals.

## 2 Methods

### 2.1 Materials

FLIPR calcium 4 assay kit was from Molecular Devices (Wokingham, UK). Fluo-4-AM, fura-2-AM and Hank's balanced salt solution (HBSS) with Ca^2+^ and Mg^2+^ were from Life Technologies (Paisley, UK). BAPTA was from Molekula (Dorset, UK). Ultragold scintillant and [2,8-^3^H]adenine were from Perkin Elmer (Buckinghamshire, UK). Ionomycin was from Apollo Scientific (Stockport, UK). Smooth muscle growth medium 2 (SMGM-2) and supplement were from Promocell (Heidelberg, Germany). Pertussis toxin (PTX) was from List Biological Laboratories (Campbell, CA, USA). Histamine dihydrochloride, carbamylcholine chloride (carbachol), (−)-isoproterenol hydrochloride, PGE_2_, 3-isobutyl-1-methylxanthine (IBMX), bradykinin acetate, Dowex 50WX4-400, alumina, imidazole, probenecid, anhydrous dimethyl sulfoxide (DMSO), pluronic F127, triton-X-100, poly-l-lysine, 8-Br-cAMP, 8-Br-cGMP, dibutyryl cAMP, KT5720 ((9*R*,10*S*,12*S*)-2,3,9,10,11,12-hexahydro-10-hydroxy-9-methyl-1-oxo-9,12-epoxy-1*H*-diindolo[1,2,3-*fg*:3′,2′,1′-*kl*]pyrrolo[3,4-*i*][1,6]benzodiazocine-10-carboxylic acid, hexyl ester), GdCl_3_, nimodipine, 2-aminoethoxyphenylborane (2-APB), ATP and acetonitrile were from Sigma (Poole, UK). 18:1 lysophosphatidic acid (LPA) was from Avanti Polar Lipids (Alabaster, AL, USA). 8-pCPT-2′-O-Me-cAMP, ESI-05 (4-methylphenyl-2,4,6-trimethylsulphone), *R*p-8-CPT-cAMPs and 6-Bnz-cAMP were from Biolog (Bremen, Germany). TCS2510 ((5*R*)-5-[(3*S*)-3-hydroxy-4-phenyl-1-buten-1-yl]-1-[6-(2*H*-tetrazol-5-yl)hexyl]-2-pyrrolidinone), H89 dihydrochloride (*N*-[2-[[3-(4-bromophenyl)-2-propenyl]amino]ethyl]-5-isoquinolinesulfonamide dihydrochloride), myristoylated protein kinase inhibitor 14–22 amide (PKI-myr), ryanodine, *trans*-Ned-19 and edelfosine were from Tocris/Biotechne (Minneapolis, MN, USA). Sulprostone was from Enzo Life Sciences (Exeter, UK). R-butaprost (free acid), NKH477 (*N*,*N*-dimethyl-(3*R*,4a*R*,5*S*,6a*S*,10*S*,10a*R*,10b*S*)-5-(acetyloxy)-3-ethenyldodecahydro-10,10b-dihydroxy-3,4a,7,7,10a-pentamethyl-1-oxo-1*H*-naphtho[2,1-*b*]pyran-6-yl ester β-alanine hydrochloride) and forskolin were from Cayman Chemicals (Ann Arbor, MI, USA). When DMSO or ethanol was used as a solvent, all related assays included solvent at the same final concentration; neither solvent, at the highest concentrations used, affected biological responses.

### 2.2 Culture of hBASMCs

Human bronchial ASM cells (hBASMC, passage 3) from three male donors (aged 11, 4 and 37 years, donors 1–3, respectively) were from Lonza (catalogue number CC-2576, Basel, Switzerland). The cells had been isolated from the major bronchi of undiseased tissue and shown to stain for α-smooth muscle actin, but not for von Willebrand Factor VIII. Cells were grown in SMGM-2 (Lonza) supplemented with fetal calf serum (5%, Sigma), and recombinant human forms of epidermal growth factor (0.5 ng·mL^−1^), basic fibroblast growth factor (2 ng·mL^−1^) and insulin (5 μg mL^−1^) (all from Promocell, Heidelberg, Germany). Cells were grown at 37 °C in humidified air containing 5% CO_2_, and passaged when they were 80–90% confluent. Cells from passages 4–10 were used for experiments. There were no obvious changes in morphology, growth rate or signaling responses within this range of passages.

### 2.3 Measurements of [Ca^2+^]_i_ in populations of hBASMCs

Two methods were used to measure [Ca^2+^]_i_ in populations of hBASMCs. For measurements using a FlexStation III plate-reader (Molecular Devices, Sunnyvale, CA, USA), hBASMCs were seeded into 96-well plates (10^4^ cells per well). After about 4 days, when the cells were confluent, the medium was replaced with SMGM-2 without serum or growth factor supplements, and the cells were used after a further 24 h. This period in serum-free medium increased by about 2-fold the amplitude of the increases in [Ca^2+^]_i_ evoked by histamine (results not shown). Cells were washed with HEPES-buffered saline (HBS: 135 mM NaCl, 5.9 mM KCl, 1.2 mM MgCl_2_, 1.5 mM CaCl_2_, 11.6 mM HEPES, 11.5 mM d-glucose, pH 7.3), and loaded with fluo-4 by incubation with fluo-4-AM (2 μM) in HBS (100 μL per well) containing pluronic F127 (0.02%, v/v) and probenecid (2.5 mM). After 1 h at 20 °C, the medium was replaced with HBS (100 μL per well) containing probenecid (2.5 mM). After a further incubation at 20 °C for 1 h, the medium was replaced with HBS (60–80 μL per well) or nominally Ca^2+^-free HBS, and the cells were used immediately for experiments at 20 °C. In some experiments, BAPTA (2.5 mM) was added to HBS during the recording; this reduced the free [Ca^2+^] of the HBS to ∼120 nM without affecting the pH. Drug additions (20 μL at 4 or 5 times the final concentration) were added automatically. Fluorescence from fluo-4 (excitation 485 nm, emission 525 nM) was recorded at 1.44-s intervals using Softmax Pro 5.4 (Molecular Devices). Fluorescence was calibrated to [Ca^2+^]_i_ from:

$${\left[{\text{Ca}}^{2+}\right]}_{\text{i}}={\text{K}}_{\text{D}}^{\text{Ca}}\times \left(\frac{\text{F}-{\text{F}}_{\text{min}}}{{\text{F}}_{\text{max}}-\text{F}}\right)$$

where, ${\text{K}}_{\text{D}}^{\text{Ca}}$ is the equilibrium dissociation constant of fluo-4 for Ca^2+^ (345 nM) [29], F is the recorded fluorescence, and F_min_ and F_max_ are the fluorescence values recorded after addition of triton-X-100 (0.1%, v/v) with either BAPTA (10 mM, F_min_) or CaCl_2_ (10 mM, F_max_). Although treatment with triton-X-100 releases fluo-4 from cells into the medium, the fluo-4 fluorescence is captured with the same efficiency whether it is trapped within cells or dispersed within the small volume of the wells (unpublished observations). F_max_ was determined for each well at the end of an experiment, and the average value was used for each column of 8 wells. F_min_ was determined from parallel wells on each plate.

For measurements of [Ca^2+^]_i_ using an FDSS 7000 FLIPR (Hamamatsu), hBASMCs were seeded into 384-well plates (8000 cells per well) in 20 μL of SMGM-2 containing 5% serum. After 24 h, the medium was replaced with 15 μL of serum-free SMGM-2, and after a further 6 h the cells were loaded with Ca^2+^ indicator by addition of FLIPR calcium 4 assay kit (Molecular Devices) supplemented with probenecid (2.5 mM). The exact composition of this ‘no-wash’ indicator kit is not disclosed by the manufacturer, but it contains fluo-4-AM and components that reduce background fluorescence. The manufacture’s stock solution was diluted 10-fold into HBSS containing BSA (0.1%, w/v) and HEPES (20 mM); 5 μL of this solution was then added to each well (containing 15 μL of serum-free SMGM-2). After 2 h at 37 °C in humidified air containing 5% CO_2_, the plate was used for experiments at 20 °C. Most additions (5 μL) were prepared in HBSS supplemented with HEPES (20 mM) and BSA (0.1%, w/v). For more prolonged incubations, drugs were diluted in the initial loading medium (to avoid changes in dye-loading during the ‘no wash’ protocol). Fluorescence signals (excitation at 480 nm, emission at 540 nm) were calibrated to [Ca^2+^]_i_ after measurement of F_min_ and F_max_ uniquely for each well, using a ${\text{K}}_{\text{D}}^{\text{Ca}}$ = 345 nM.

All concentration-effect relationships were determined by addition of different drug concentrations to individual wells in the same multi-well plate, rather than by sequential additions to the same well.

### 2.4 Measurements of intracellular cAMP by [^3^H]-adenine labeling

hBASMCs in 24-well plates (50,000 cells per well) were grown to confluence. The medium was then replaced with serum-free SMGM-2, and after 24 h this was supplemented with [^3^H]-adenine (1 μCi per well, 18.4 Ci·mmol^−1^). After 2 h at 37 °C in humidified air with 5% CO_2_, the medium was removed, and the cells were washed twice with HBS. The cells were stimulated at 20 °C in HBS. Incubations were terminated by removal of the medium, addition of ice-cold trichloroacetic acid (5%, 1 mL) and rapid freezing. This protocol ensured that only intracellular [^3^H]-cAMP was detected. [^3^H]-adenine nucleotides were separated by column chromatography [30], and the activity was determined by liquid scintillation counting using Ultra-gold scintillant. Results are presented as [^3^H]-cAMP activity as a percentage of the sum of the activities of the fractions containing [^3^H]-cAMP, [^3^H]-ATP, [^3^H]-ADP and [^3^H]-AMP; henceforth, reported as [^3^H]-cAMP (%).

### 2.5 Measurements of intracellular cAMP by mass spectrometry

These methods were modified from [31]. Confluent cultures of hBASMCs in 48-well plates were serum-deprived (6–24 h) and the medium was then replaced with HBSS (300 μL) containing HEPES (5 mM) and BSA (0.1%, w/v). Cells were stimulated at 20 °C, and reactions were terminated by aspiration of the medium and addition of acetonitrile (170 μL) containing dibutyryl cAMP (0.5 μM, to provide an internal standard). The plates were centrifuged (1500 x*g*, 15 min, 4 °C), supernatants (158 μL) were transferred to a 96-well, glass-coated plate (Thermo Scientific), and aqueous NH_4_HCO_3_ (pH 9.4, 93 μL) was added to each sample. After mixing and centrifugation (1500 x*g*, 15 min, 4 °C), samples were stored at 4 °C before analysis.

Samples were analyzed by liquid chromatography tandem mass spectrometry (LC–MS/MS) using a Waters Acquity UPLC and a Sciex 5500 mass spectrometer equipped with an electrospray ionization source. ATP, ADP, AMP and cAMP were analyzed using a ZIC-pHILIC polymeric column (5-μm particle size, 5.0 × 2.1 mm) maintained at 35 °C. Calibration standards were prepared in the lysis medium containing the internal standard, dibutyryl cAMP (0.5 μM). The injector was maintained at 4 °C and injection volumes were 5 μL. The mobile phase comprised solvents A (20% acetonitrile, 80% aqueous NH_4_HCO_3_, pH 9.4) and B (100% acetonitrile). The mobile phase (0.4 mL·min^−1^) was 40% A (0.2 min), then a linear gradient from 40% to 100% A (0.8 min), followed by 100% A (1 min). The gradient was returned to the initial conditions over 0.5 min, and maintained for a further 1.5 min. Samples were detected using multiple reaction monitoring in negative ion mode using the following parent-to-daughter mass transitions: cAMP *m*/*z* 327.9 → 133.9 (DP −100 V, CE −33 eV), AMP *m*/*z* 345.9 → 134.0 (DP −100 V, CE −50 eV), ADP *m*/*z* 426.0 → 134.0 (DP −100 V, CE −30 eV), ATP *m*/*z* 505.9 → 408.0 (DP −100 V, CE −35 eV), and internal standard dibutyryl cAMP *m*/*z* 468.1 → 175.0 (DP −100 V, CE −35 eV).

### 2.6 Data and statistical analysis

Concentration-effect relationships were fitted to logistic equations for each experiment using GraphPad Prism (version 5, GraphPad Software, La Jolla, CA, USA), from which half-maximally effective drug concentrations (EC_50_), maximal responses, and Hill slopes were determined. For statistical analyses, maximal responses, Hill slopes and pEC_50_ (-logEC_50_) or pIC_50_ (-log of the half-maximal inhibitory concentration, IC_50_) values determined for individual experiments were pooled for statistical analysis. Two-tailed Student’s *t*-tests or one-way ANOVA with Bonferroni’s or Dunnett’s multiple comparison tests were used as appropriate. *P <* 0.05 was considered significant. Results are reported as mean ± SEM with *n* indicating the number of independent experiments (ie performed with different culture plates on different days, and usually with all reagents independently prepared). Most statistical analyses used GraphPad Prism (version 5).

## 3 Results

### 3.1 GPCRs evoke Ca^2+^ signals in hBASMCs through IP_3_Rs

**Fig. 1:**
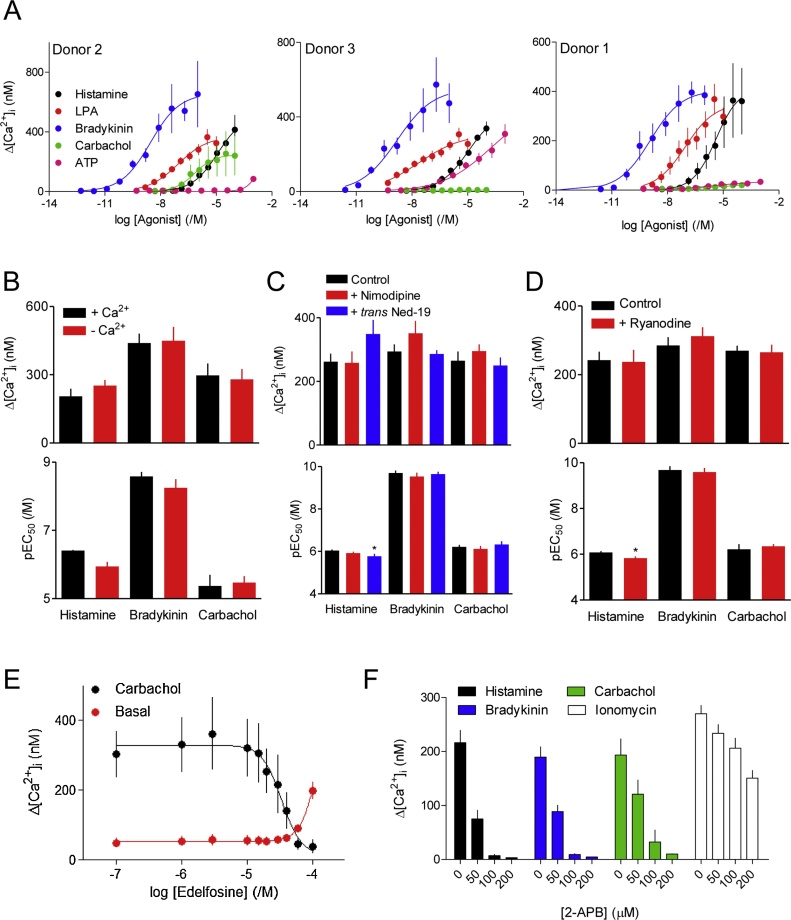
GPCRs stimulate increases in [Ca^2+^]_i_ in hBASMCs through activation of PLC and IP_3_Rs. A, Populations of fluo-4-loaded hBASMCs in 384-well plates were stimulated with the indicated drug concentrations in HBSS. Peak increases in [Ca^2+^]_i_ are shown (Δ[Ca^2+^]_i_) as means ± SEM for cells from donors 1, 2 and 3 (*n* = 4, 3 and 3, respectively). B, Effects of histamine, bradykinin and carbachol on Δ[Ca^2+^]_i_ and the sensitivity to each (pEC_50_) in either HBS or Ca^2+^-free HBS (2.5 mM BAPTA added 37 s before the stimulus). Cells were from donor 1 for histamine and bradykinin (*n* = 3) and from donor 2 for carbachol (*n* = 4). C, D, Effects of nimodipine (10 μM, 5 min), *trans* Ned-19 (1 μM, 5 min) or ryanodine (50 μM, 5 min) on the Ca^2+^ signals evoked by the indicated stimuli in HBSS. Results (B-D) show means ± SEM, *n* = 7 (histamine, donor 1), *n* = 4 (bradykinin, donor 1) and *n* = 3 (carbachol, donor 2). ^*^P < 0.05, one-way repeated ANOVA with Dunnett’s test (C) or paired two-tailed Student’s *t*-test (D), each relative to control. E, Effects of pre-incubation (30 min) with the indicated concentrations of edelfosine on basal [Ca^2+^]_i_ and the peak increases in [Ca^2+^]_i_ evoked by carbachol (10 μM). Results (mean ± SEM, *n* = 3) are from donor 2. F, Effects of the indicated concentrations, of 2-APB added 5 min before histamine (10 μM, *n* = 7), carbachol (10 μM, *n* = 3) or bradykinin (1 nM, *n* = 4) in HBSS, or to ionomycin (1 μM, *n* = 7) added in Ca^2+^-free HBSS to determine the Ca^2+^ content of the intracellular stores. Results are from donors 1 and 2.

Stimuli of several GPCRs reported to be expressed in ASM evoked increases in [Ca^2+^]_i_ in hBASMCs (Fig. 1A). Cells from all three donors responded to histamine, LPA and bradykinin, but cells from only one donor responded robustly to ATP (donor 3) and cells from only one other donor responded robustly to carbachol (donor 2) (Fig. 1A). The response to carbachol was unexpected because although native hBASMCs express M_2_ and M_3_ muscarinic receptors [12], their expression is usually lost when cells are cultured (see Section 1). Our identification of functional muscarinic receptors provides the first opportunity to examine the effects of the most important physiological stimulus for contraction in cultured hBASMCs.

The peak increases in [Ca^2+^]_i_ evoked by histamine, bradykinin or carbachol were unaffected by removal of extracellular Ca^2+^, confirming that the initial response was entirely mediated by release of Ca^2+^ from intracellular stores (Fig. 1B). The responses were also unaffected by block of L-type Ca^2+^ channels with nimodipine (Fig. 1C). In a parallel experiment with single fura-2-loaded hBASMCs, replacing extracellular Na^+^ with K^+^ (140 mM) to evoke depolarization caused a detectable increase in [Ca^2+^]_i_ in 29 of 44 cells (from a single experiment). This response was reversibly inhibited by nimodipine (10 μM, 5 min): the peak increase in [Ca^2+^]_i_ recorded from all 44 cells was 50 ± 11 nM and 20 ± 14 nM in the absence and presence of nimodipine, respectively (mean ± SD from a single experiment, *P <* 0.05, Student’s *t*-test). Neither ryanodine to inhibit ryanodine receptors (RyR) (Fig. 1D) nor *trans* Ned-19 to inhibit two-pore channels (TPC) [32,but see reference 33] (Fig. 1C) substantially affected the Ca^2+^ signals evoked by histamine, bradykinin or carbachol, although the sensitivity to histamine was slightly reduced by both inhibitors. The concentrations of the inhibitors used were shown by others to effectively inhibit their targets [see references in 34] A lack of response to caffeine (data not shown) and the insensitivity of most responses to ryanodine (Fig. 1D) may reflect a loss of functional RyRs during culture of hBASMCs, as noted previously for other smooth muscle cells [34]. However, even in human lung slices, which do express RyRs, histamine-evoked Ca^2+^ signals were unaffected by inhibition of RyRs [19].

Edelfosine, an inhibitor of PLC [35], caused a concentration-dependent inhibition of the responses evoked by carbachol (Fig. 1E), histamine and bradykinin. The pIC_50_ values for inhibition by edelfosine of the Ca^2+^ signals evoked by histamine (10 μM), bradykinin (1 nM) and carbachol (10 μM) were 4.64 ± 0.13, 4.47 ± 0.06 and 4.40 ± 0.04, respectively (*n* = 3). There are no selective and effective membrane-permeant inhibitors of IP_3_Rs [36]. 2-APB inhibits IP_3_Rs, but it also modulates store-operated Ca^2+^ entry, and it inhibits the Ca^2+^ pump that mediates Ca^2+^ uptake into the ER [37]. The results shown in Fig. 1F demonstrate that under conditions where Ca^2+^ entry does not contribute to the GPCR-evoked Ca^2+^ signals (Fig. 1B), 2-APB abolished the increases in [Ca^2+^]_i_ evoked by histamine, bradykinin and carbachol. 2-APB also reduced the Ca^2+^ content of the intracellular stores (assessed by addition of ionomycin in Ca^2+^-free HBS), but this effect was less substantial and required higher concentrations of 2-APB than the inhibition of GPCR-evoked Ca^2+^ signals (Fig. 1F).

**Fig. 2:**
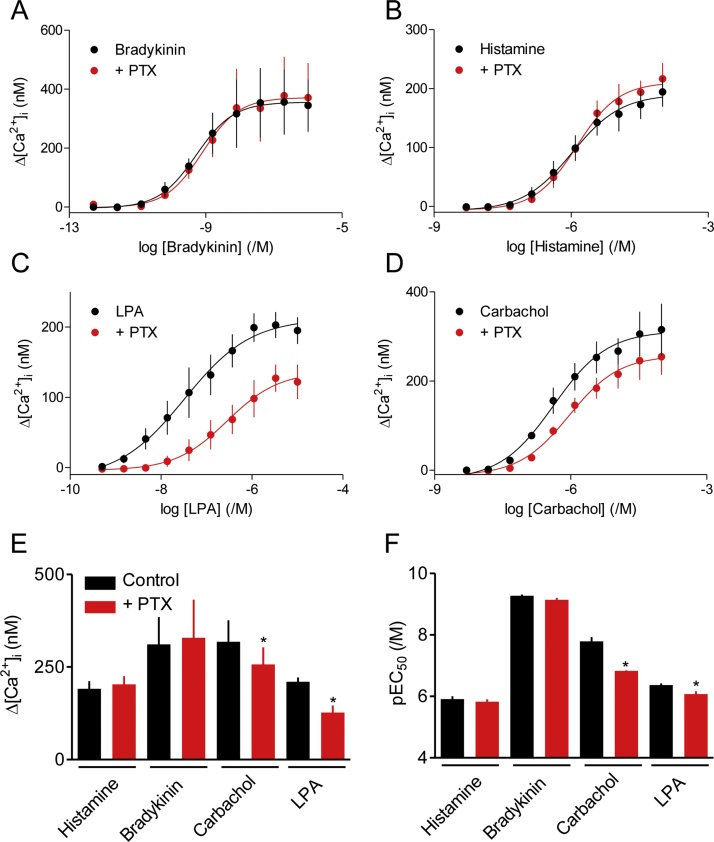
Pertussis toxin selectivity attenuates the Ca^2+^ signals evoked by LPA and carbachol. A-D, Effects of pre-treatment with pertussis toxin (PTX, 100 ng·mL^−1^, 24 h) on the peak increases in [Ca^2+^]_i_ evoked by the indicated stimuli. Results are from cells derived from donors 1 and 2 (*n* = 3 for *B* and *C*; *n* = 4 for A and D). E, F, Summary results. ^*^P < 0.05, paired Student’s *t*-test, relative to control.

Pre-treatment of hBASMCs with pertussis toxin (PTX) had no effect on the Ca^2+^ signals evoked by histamine or bradykinin, but it significantly reduced both the maximal amplitude of the Ca^2+^ signals evoked by carbachol and LPA and their sensitivity to these stimuli (Fig. 2). The incomplete block of responses to carbachol and LPA by PTX is unlikely to result from incomplete modification of G_i_ proteins, because in parallel experiments the same treatment with PTX abolished the inhibition of AC activity by carbachol, probably acting via M_2_ muscarinic receptors [12] (data not shown).

The results so far demonstrate that histamine, bradykinin, carbachol, ATP and LPA can evoke Ca^2+^ signals in hBASMCs. The initial responses are likely to be mediated by Ca^2+^ release through IP_3_Rs after stimulation of PLCβ by G_q/11_. In addition, release of Gβγ subunits from G_i_ contributes to responses evoked by carbachol, and more so to the Ca^2+^ signals evoked by LPA. The results with carbachol are consistent with evidence from human lung tissue showing that M_3_ receptors mediate most carbachol-evoked contraction, with lesser [12] or undetectable [38] contributions from M_2_ receptors.

### 3.2 Isoproterenol inhibits histamine-evoked Ca^2+^ signals through cAMP and PKA

**Fig. 3:**
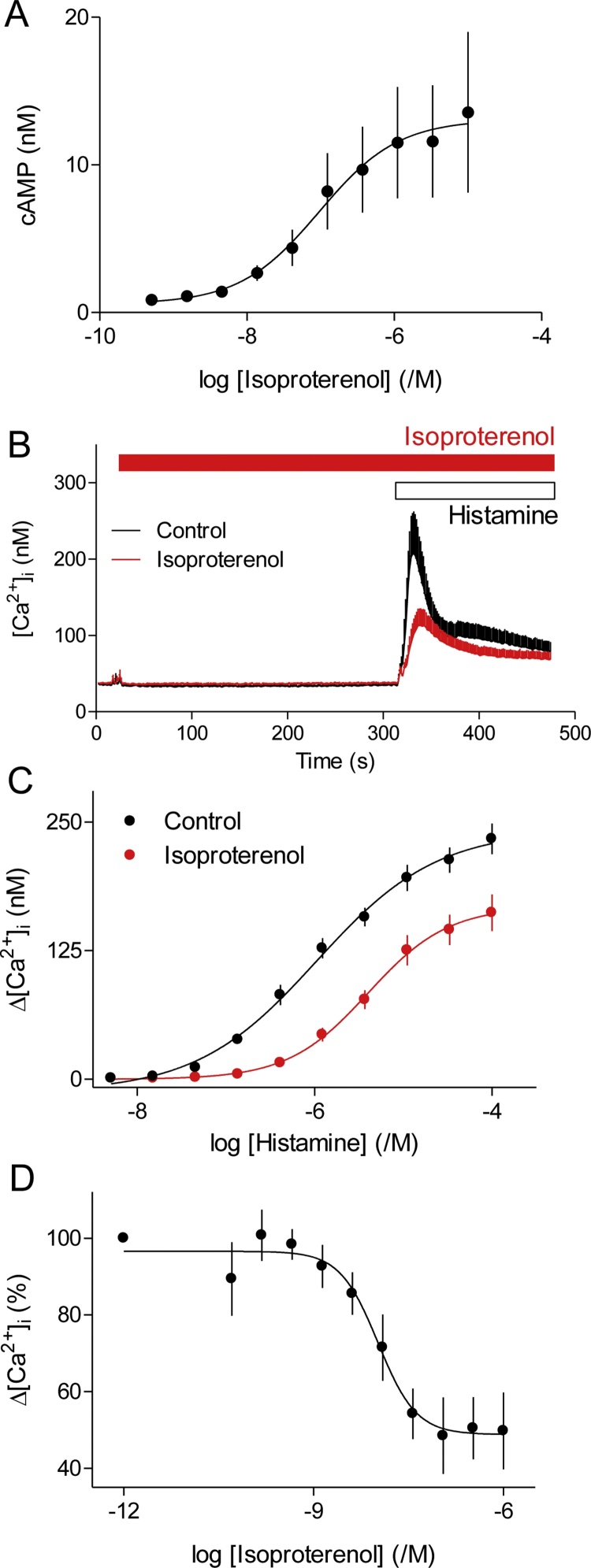
Inhibition of histamine-evoked Ca^2+^ signals by isoproterenol. A, Effects of isoproterenol (5 min) on intracellular cAMP concentrations in hBASMCs. Results are from MS analyses, *n* = 3. B, Typical traces from populations of fluo-4-loaded hBASMCs stimulated in HBS with histamine alone (10 μM, black trace) or after pre-incubation with isoproterenol (10 μM, 5 min, red trace) (*n* = 6). C, Summary results from similar experiments performed in HBSS (*n* = 7) show Δ[Ca^2+^]_i_ evoked by histamine alone or after treatment with isoproterenol. D, Concentration-dependent effects of isoproterenol (added 5 min before histamine) on Δ[Ca^2+^]_i_ evoked by histamine (10 μM) in HBSS. Results, are expressed as percentages of the matched control response without isoproterenol (*n* = 4). Results are from donor 1 (A, C and D) or donors 1 and 2 (B).

Activation of β-adrenoceptors with isoproterenol stimulated a concentration-dependent accumulation of intracellular cAMP within hBASMCs (Fig. 3A). Isoproterenol also inhibited histamine-evoked Ca^2+^ signals by significantly reducing both the maximal amplitude of the peak increase in [Ca^2+^]_i_ (from 242 ± 15 nM to 168 ± 19 nM, *n* = 7) and the sensitivity to histamine (pEC_50_ = 5.98 and 5.37) (Fig. 3B, C). Histamine-evoked Ca^2+^ signals were more sensitive to isoproterenol (pIC_50_ = 8.09 ± 0.21, *n* = 4) than was cAMP accumulation (pEC_50_ = 6.88 ± 0.39, *n* = 3) (Fig. 3A, D).

**Fig. 4:**
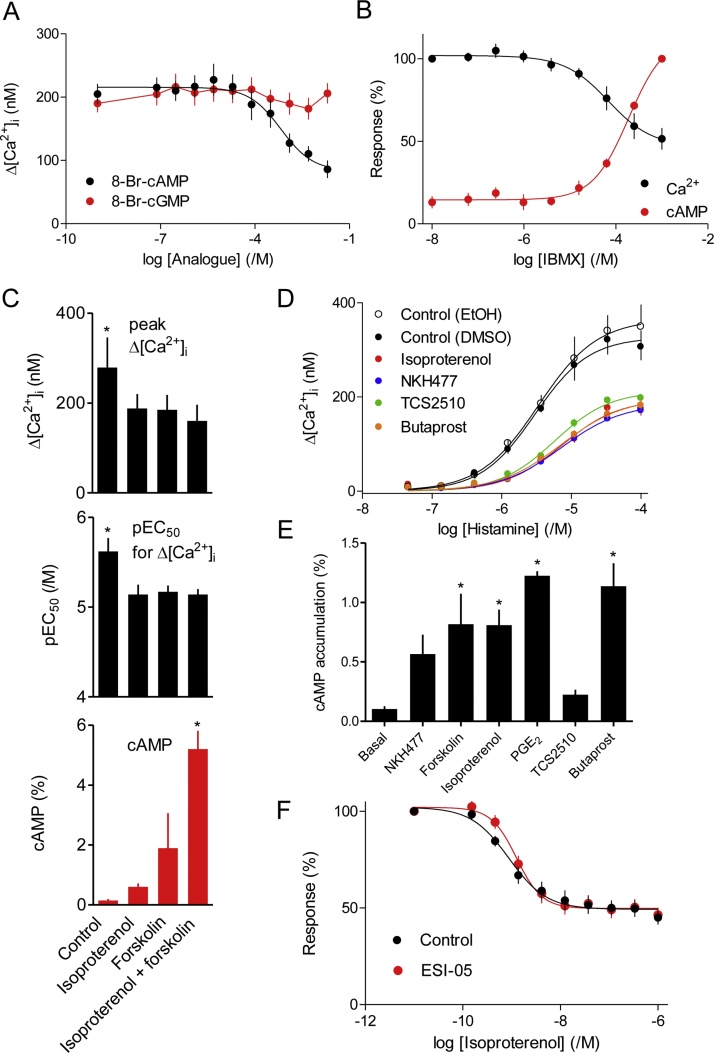
Isoproterenol inhibits histamine-evoked Ca^2+^ signals through cAMP. A, Peak increases in [Ca^2+^]_i_ evoked by histamine (10 μM) after pre-treatment with the indicated concentrations of 8-Br-cAMP or 8-Br-cGMP (20 min) (*n* = 4). B, Effects of the indicated concentrations of IBMX (20 min) on the intracellular concentration of cAMP (measured by MS) and the peak increase in [Ca^2+^]_i_ evoked by histamine (10 μM). Results are expressed as percentages of the Δ[Ca^2+^]_i_ evoked by histamine alone (*n* = 6, donor 1) or as percentages of the cAMP concentration determined with the maximal concentration of IBMX (1 mM) (*n* = 5, donor 1). C, Effects of pre-incubation (30 min) with isoproterenol (10 μM), forskolin (10 μM) or both on the peak increase in [Ca^2+^]_i_ evoked by histamine in HBSS, and their sensitivity to histamine (pEC_50_) (donor 1, *n* = 4). Parallel experiments show effects of the same treatments on intracellular cAMP accumulation determined after ^3^H-adenine-labeling of cells in HBS (donor 1, *n* = 3). ^*^P < 0.05, one-way repeated measures ANOVA with Dunnett’s test, relative to response evoked in the presence of isoproterenol. D, Peak increases in [Ca^2+^]_i_ evoked by the indicated concentrations of histamine in HBS after pre-treatment (5 min) with solvents (DMSO or EtOH), isoproterenol (10 μM), NKH477 (10 μM), TCS 2510 (1 μM) or butaprost (10 μM) (*n* = 4). E, Intracellular cAMP accumulation in hBASMCs stimulated for 5 min in HBS with NKH477 (10 μM), forskolin (10 μM), isoproterenol (10 μM), PGE_2_ (10 μM), TCS 2510 (1 μM) or butaprost (10 μM). Results ([^3^H]-cAMP, %, see Methods) are from donor 1 (*n* = 6-8), but were confirmed in donor 2. ^*^P < 0.05, one-way repeated measures ANOVA with Dunnett’s test, relative to basal. F, Effects of ESI-05 (25 μM, 30 min) in HBS on the Ca^2+^ signals evoked by histamine (10 μM) added 1 min after the indicated concentrations of isoproterenol. Results are expressed as percentages of matched responses to histamine in the absence of isoproterenol (donors 1 and 2, *n* = 9).

The inhibition of histamine-evoked Ca^2+^ signals by isoproterenol was mimicked by a membrane-permeant analogue of cAMP, 8-Br-cAMP (pIC_50_ = 3.32 ± 0.16, *n* = 6), but not by 8-Br-cGMP (Fig. 4A). Inhibition of cyclic nucleotide phosphodiesterases with IBMX also caused an accumulation of intracellular cAMP and an inhibition of histamine-evoked Ca^2+^ signals (Fig. 4B). Direct activation of AC by forskolin (Fig. 4*C*) or its more water-soluble analogue NKH477 (Fig. 4D) also mimicked the effect of isoproterenol. Neither isoproterenol nor forskolin (10 μM, 5 min) affected the Ca^2+^ content of the intracellular stores, assessed by addition of ionomycin in Ca^2+^-free HBS (results not shown). The maximal inhibitory effects of forskolin and isoproterenol on histamine-evoked Ca^2+^ signals were similar and no larger with both stimuli together (Fig. 4C), although their combined effects on cAMP accumulation were larger than with either stimulus alone (Fig. 4C, E). These results suggest that either stimulus can evoke formation of more cAMP than needed to maximally inhibit the histamine-evoked Ca^2+^ signals, consistent with our evidence that the inhibition of Ca^2+^ signals is more sensitive than the formation of cAMP to isoproterenol (Fig. 1A and 3D). These results are consistent with cAMP preceding inhibition of Ca^2+^ signals in the signaling pathway [39], and with maximal activation by isoproterenol generating more cAMP than required to maximally inhibit the Ca^2+^ signals. Our results confirm those from human lung slices, where histamine-evoked Ca^2+^ signals and contractions were attenuated by formoterol [19], and they extend them by demonstrating that the effects of β_2_-adrenoceptors are entirely mediated by cAMP.

**Fig. 5:**
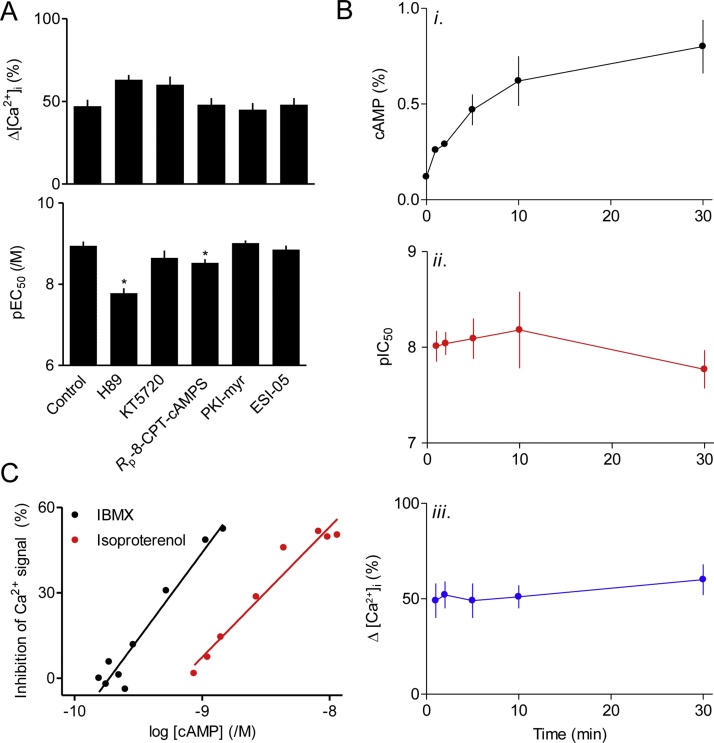
Compartmentalized cAMP inhibits histamine-evoked Ca^2+^ signals. A, Effects of pre-treatment (30 min) with the indicated inhibitors and then isoproterenol (1 min) on the Ca^2+^ signals evoked by histamine (10 μM). Results (donors 1 and 2, *n* = 9) show the peak Ca^2+^ signals (as percentages of matched responses to histamine without isoproterenol) and their sensitivity to inhibition by isoproterenol (pIC_50_). ^*^P < 0.05, one-way repeated measures ANOVA with Dunnett’s test, relative to control. B, Effects of varying the duration of the incubation with isoproterenol (10 μM) on cAMP accumulation (i) and the peak Ca^2+^ signals evoked by histamine (10 μM) (ii and iii). Accumulation of intracellular cAMP was measured after ^3^H-adenine labeling ([^3^H]-cAMP, %). Results for Δ[Ca^2+^]_i_ show the peak response as a percentage of that evoked by histamine alone (ii) and the pIC_50_ value for isoproterenol (iii). (*n* = 4). C, Relationship between intracellular cAMP (determined by MS) and the inhibition of Ca^2+^ signals evoked by histamine (10 μM) in cells where the increase in cAMP was evoked by incubation with different concentrations of IBMX (20 min) or isoproterenol (5 min). Each point includes data from 5 (IBMX) or 3 (isoproterenol) MS determination of cAMP associated with 6 (IBMX) or 4 (isoproterenol) measurements of [Ca^2+^]_i_. Results (B and C) are from cells from donor 1.

While our work was in progress, the first direct evidence confirming a role for PKA in mediating the effects of β_2_-adrenoceptors on Ca^2+^ signals and relaxation of cultured smooth muscle from human trachea and bronchi was published [5]. The authors demonstrated that stable expression of a peptide inhibitor of PKA (PKI) abolished the inhibition of histamine-evoked Ca^2+^ signals by isoproterenol, knockdown of Epacs 1 and 2 had no effect on the inhibition by isoproterenol of the histamine-stimulated phosphorylation of myosin light chain 2, and nor did an Epac-selective cAMP analogue mimic the effect of isoproterenol. Our results are consistent with their conclusion that Epacs do not contribute to the inhibition of histamine-evoked Ca^2+^ signals by isoproterenol. Pre-treatment of hBASMCs with a cAMP analogue selective for PKA, 6-Bnz-cAMP (500 μM, 20 min) [40], reduced the amplitude of the Ca^2+^ signals evoked by histamine (10 μM) to 54 ± 4% of these recorded from paired controls (*n* = 5), whereas the Epac-selective analog 8-pCPT-2′-*O*-Me-cAMP (300 μM) had no effect (103 ± 8%). Two of the commonly used antagonists of Epacs (ESI-09 and HJC0197) [41] have intolerable off-target effects [42,43]. However, an Epac-2 inhibitor, ESI-05 [41], had no effect on the concentration-dependent inhibition of histamine-evoked Ca^2+^ signals by isoproterenol (Figs. Fig. 4F and Fig. 5A). Our results with inhibitors of PKA, in keeping with similar published approaches [44], were inconclusive (Fig. 5A). Inhibitors expected to interact with the ATP-binding site of PKA (H89 and KT5720), its cAMP-binding site (*R*_p_-8-CPT-cAMPS) or its peptide-binding site (PKI-myr) had no significant effect on the inhibition of histamine-evoked Ca^2+^ signals by a maximally effective concentration of isoproterenol (Fig. 5A). H89 significantly reduced the sensitivity to isoproterenol, but that may be due to it being a competitive antagonist of β-adrenoceptors [45]. *R*_p_-8-CPT-cAMPS also caused a significant decrease in the sensitivity to isoproterenol, but the effect was small (Fig. 5A). Neither KT5720 nor PKI-myr significantly affected the sensitivity to isoproterenol. Both we and others have failed to achieve effective inhibition of PKA in intact smooth muscle cells with these inhibitors [[5],^43^,^44^]. However, in light of the recently published work we suggest that PKA probably mediates most relaxant effects of isoproterenol in human ASM [5] and it is therefore likely also to mediate the effects of isoproterenol on histamine-evoked Ca^2+^ signals. That conclusion is also consistent with recent analyses of human aortic smooth muscle, where selective inhibition of histamine-evoked Ca^2+^ signals by PGE_2_ was shown to be mediated by PKA [43].

### 3.3 Isoproterenol signals to Ca^2+^ signals through compartmentalized cAMP

During sustained incubation with isoproterenol, cAMP continued to accumulate for at least 30 min, such that the stimulated cAMP concentration was 2.8-fold higher after 30 min than after 1 min (Fig. 5Bi). However, the inhibition of Ca^2+^ signals was similar when hBASMCs were pre-incubated with isoproterenol for intervals between 1 and 30 min before addition of histamine (Fig. 5Bii, Biii). Hence, even though cAMP continued to accumulate long after the first minute of stimulation with isoproterenol, neither the maximal inhibition of histamine-evoked Ca^2+^ signals nor their sensitivity (pIC_50_) to isoproterenol was increased by prolonging the incubation (Fig. 5B). Since maximal activation of β_2_-adrenoceptors provides more cAMP than needed to maximally inhibit histamine-evoked Ca^2+^ signal, it is unsurprising that prolonged incubation with a maximally effective concentration of isoproterenol caused no further inhibition of Ca^2+^ signals. However, when cAMP entirely mediates the effects of isoproterenol (Fig. 4), it is surprising that the sensitivity of histamine-evoked Ca^2+^ signals to isoproterenol is unaffected by prolonged incubations during which more intracellular cAMP accumulates (Fig. 5B). Inhibition of Ca^2+^ signals by β_2_-adrenoceptors cannot, therefore, be mediated by cAMP uniformly distributed throughout the cytosol.

Whereas GPCRs may locally deliver cAMP at high concentrations to targets within signaling junctions [28 and references therein], this is less likely for cAMP accumulated after addition of IBMX, a non-selective inhibitor of cyclic nucleotide phosphodiesterases (Fig. 4B). We therefore compared the relationship between intracellular cAMP and inhibition of histamine-evoked Ca^2+^ signals, for cAMP responses evoked by IBMX or isoproterenol. For matched Ca^2+^ signals, the inhibition evoked by isoproterenol was associated with ∼5.4-fold higher concentrations of intracellular cAMP than for IBMX (Fig. 5C). These results again suggest that histamine-evoked Ca^2+^ signals are not regulated by globally distributed cAMP.

### 3.4 PGE_2_ inhibits histamine-evoked Ca^2+^ signals through both EP_2_ and EP_4_ receptors

**Fig. 6:**
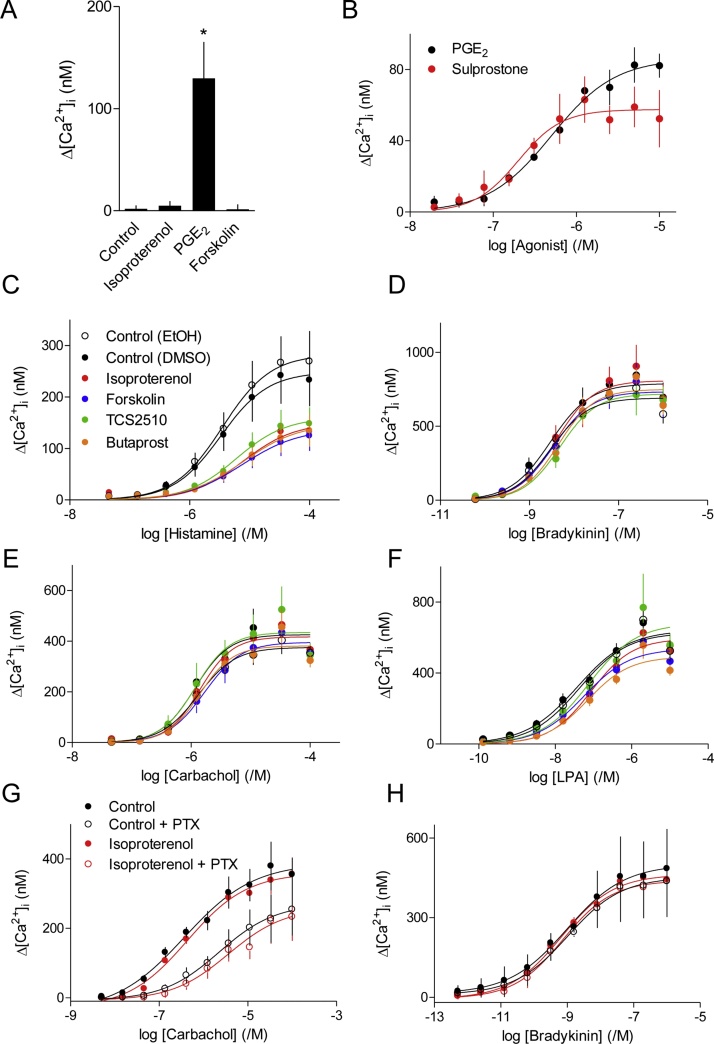
Ca^2+^ signals evoked by different GPCRs differ in their susceptibility to inhibition by cAMP. A, Peak increases in [Ca^2+^]_i_ evoked by isoproterenol (10 μM), PGE_2_ (10 μM) or forskolin (10 μM) in Ca^2+^-free HBS (*n* = 7 from donors 1 and 2). ^*^P < 0.05, one-way ANOVA with Dunnett's test, relative to control. B, Peak increases in [Ca^2+^]_i_ evoked by PGE_2_ or sulprostone in Ca^2+^-free HBS (BAPTA added 37 s before the stimuli) (*n* = 3 from donors 1 and 2). C-F, hBASMCs in HBS were pre-treated (5 min) with isoproterenol (10 μM), forskolin (10 μM), butaprost (10 μM), TCS2510 (1 μM) or solvents, and then stimulated with the indicated concentrations of histamine (C), bradykinin (D), carbachol (E) or LPA (F). The code in C applies to panels C-F. Results show peak increases in [Ca^2+^]_i_ evoked by the final stimulus from 6 independent experiments from donors 1 and 2 (C, D and F), and from 3 independent experiments with donor 2 (E). (G, H) Similar analyses of cells in HBS after treatment with pertussis toxin (PTX, 100 ng·mL^−1^, 24 h). The code in G applies also to H. Results are from 3 independent experiments from donor 2 (G) and donors 1 and 2 (H).

In hBASMCs, PGE_2_ stimulated cAMP accumulation (Fig. 4E) but, unlike isoproterenol or forskolin, PGE_2_ directly evoked a significant increase in [Ca^2+^]_i_ (Fig. 6A). This response was probably mediated by EP_3_ receptors because sulprostone, a selective agonist of G_i_-coupled EP_3_ receptors [46], also evoked an increase in [Ca^2+^]_i_ (Fig. 6B).

In subsequent experiments, butaprost and TCS2510 were used to selectively stimulate EP_2_ and EP_4_ receptors, respectively. Both receptors are known to stimulate Gs and thereby AC activity [47,48]. Neither butaprost nor TCS2510 evoked an increase in [Ca^2+^]_i_ (results not shown), but both stimulated formation of cAMP and inhibited the Ca^2+^ signals evoked by histamine (Fig. 4D, E). Although a maximal concentration of TCS2510 was as effective as forskolin, NKH477, isoproterenol or butaprost in inhibiting histamine-evoked Ca^2+^ signals, it evoked far less production of cAMP (Fig. 4E). This suggests that maximal activation of EP_2_ receptors (by butaprost), like maximal activation of β-adrenoceptors (by isoproterenol), evokes formation of more cAMP than needed to cause maximal inhibition of histamine-evoked Ca^2+^ signals. Our results are consistent with evidence that human ASM express EP_2_, EP_3_ and EP_4_ receptors, that EP_3_ receptors evoke an increase in [Ca^2+^]_i_, that both EP_2_ and EP_4_ receptors stimulate accumulation of cAMP [26], and with recent evidence showing that EP_2_ receptors evoke local cAMP signals in human ASM [28]. PGE_2_ causes relaxation of histamine-contracted human airways, but conflicting reports have suggested that this is mediated entirely through EP_2_ [27] or EP_4_ receptors [22]. Our results provide the first evidence that both EP_2_ and EP_4_ receptors inhibit histamine-evoked Ca^2+^ signals in human ASM.

### 3.5 Ca^2+^ signals evoked by different GPCRs differ in their susceptibility to inhibition by cAMP

Fig. 6C–F compares the effects of activating AC directly (with forskolin) or via G_s_-coupled GPCRs (β_2_-adrenoceptors, EP_2_ or EP_4_ receptors) on the Ca^2+^ signals evoked by histamine, LPA, bradykinin or carbachol in hBASMCs. The results confirm the substantial inhibition of histamine-evoked Ca^2+^ signals by cAMP, but the Ca^2+^ signals evoked by carbachol and bradykinin were unaffected by any of the cAMP-elevating stimuli. Analyses of single fura 2-loaded cells confirmed that all cells responded to both histamine and bradykinin with an increase in [Ca^2+^]_i_ (results not shown). The differential susceptibility of the Ca^2+^ signals evoked by histamine and bradykinin to inhibition by cAMP is not therefore due to differential distribution of their receptors between cells.

Activation of M_2_ receptors by carbachol might, via G_i_, have counteracted the increases in cAMP evoked by forskolin or the G_s_-coupled GPCRs [12,38]. However, the Ca^2+^ signals evoked by carbachol or bradykinin remained insensitive to isoproterenol after treatment with pertussis toxin (Fig. 6G, H). Parallel experiments demonstrated that the treatment with PTX was sufficient to abolish the inhibition of AC activity by carbachol (results not shown) and to attenuate the Ca^2+^ signals evoked by carbachol and LPA (Fig. 2C-F). These results demonstrate that cAMP selectively inhibits the Ca^2+^ signals evoked by histamine.

## 4 Discusssion

**Fig. 7:**
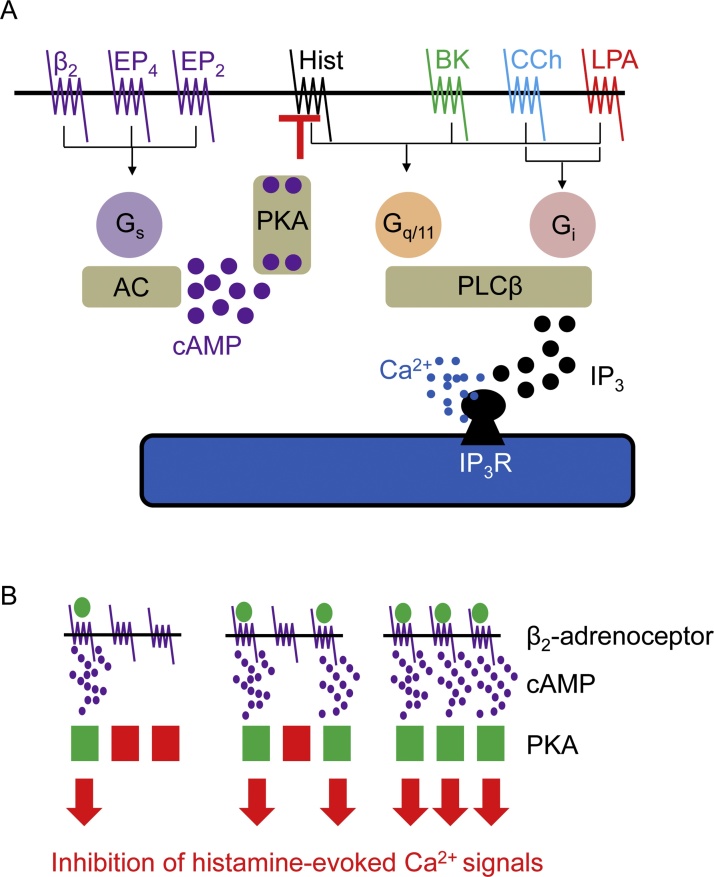
Selective inhibition of histamine-evoked Ca^2+^ signals by hyperactive cAMP junctions in human airway smooth muscle. A, Histamine (Hist), bradykinin (BK), carbachol (CCh) and LPA through their respective GPCRs stimulate PLCβ entirely through G_q/ll_ or with some contribution from G_i_. IP_3_ then stimulates Ca^2+^ release through IP_3_Rs within the sarcoplasmic reticulum. β_2_-adrenoceptors or receptors for PGE_2_ (EP_2_ and EP_4_) stimulate AC and thereby PKA, which selectively inhibits the Ca^2+^ signals evoked by histamine, perhaps through phosphorylation of H_1_ histamine receptors by PKA. B, Cyclic AMP may be delivered to PKA within ‘hyperactive’ signaling junctions, such that activation of a junction provides more than enough local cAMP to saturate the associated PKA. The junction thereby functions as a robust on-off switch. Concentration-dependent responses to β_2_-agonists are due to recruitment of these digital junctions.

Our analyses of hBASMCs show that histamine evokes cytosolic Ca^2+^ signals by stimulating PLC and release of Ca^2+^ through IP_3_Rs (Fig. 1A–D). Similar mechanisms probably underlie the Ca^2+^ signals evoked by carbachol, bradykinin and LPA (Fig. 1B–D). The four stimuli do, however, differ in the extent to which they regulate PLC exclusively through G_q/11_ (histamine and bradykinin) or with some contribution from Gi (LPA, and to a lesser extent carbachol) (Figs. 2, 6G, H and 7A). These results concur with those from human lung slices, where contractions evoked by carbachol or histamine were substantially attenuated by inhibitors of G_q/11_ [49], although the specificity of one of the inhibitors (UBO-QIC) has been challenged [50].

The Ca^2+^ signals evoked by histamine were attenuated by stimulation of β_2_-adrenoceptors, consistent with results from human lung slices where formoterol caused a long-lasting inhibition of histamine-evoked Ca^2+^ oscillations [19]. In our analyses, the inhibition was mimicked by stimulation of EP_2_ or EP_4_ receptors, 8-Br-cAMP, direct activation of AC, or by inhibition of cyclic nucleotide PDEs (Fig. 3, Fig. 4, Fig. 5, Fig. 6). These results and the non-additive inhibition of Ca^2+^ signals by maximally effective concentrations of forskolin and isoproterenol (Fig. 4C) establish that inhibition of histamine-evoked Ca^2+^ signals by β_2_-adrenoceptors is entirely mediated by cAMP. The inhibition is not mediated by activation of epacs (Fig. 4F and 5A), but our attempts to demonstrate a need for PKA were thwarted by ineffective inhibitors (Fig. 5A)[for further discussion see [5], 43]. However while our work was in progress, inhibition of histamine-evoked Ca^2+^ signals by isoproterenol was shown to be prevented by viral infection with a peptide inhibitor of PKA [5]. Hence, we suggest that in hBASMCs inhibition of histamine-evoke Ca^2+^ signals by β-adrenoceptors is entirely mediated by cAMP and PKA (Fig. 7A).

The inhibition of GPCR-evoked Ca^2+^ signals by cAMP was selective for histamine. The Ca^2+^ signals evoked by bradykinin and carbachol were insensitive to cAMP, while cAMP caused only a modest and inconsistent inhibition of responses to LPA (Figs. 6C—H and 7A). Isoproterenol can cause relaxation of airways in human lung slices contracted with carbachol [12], but the contractions evoked by muscarinic receptors are more resistant to the relaxant effects of β_2_-adrenoceptors than the contractions evoked by histamine [38,[51], [52], [53]]. These results are consistent with our findings, and suggest that a reduction in the sensitivity of the contractile apparatus to Ca^2+^ by cAMP may reduce contractions evoked by all contractile stimuli [19], while the response to histamine is further reduced by attenuation of the Ca^2+^ signals. Selective inhibition of histamine-evoked Ca^2+^ signals by PKA suggests a target close to the histamine H_1_ receptor, and perhaps the receptor itself [see discussion in reference 43] (Fig. 7A).

Although cAMP entirely mediates the inhibition of histamine-evoked Ca^2+^ signals by β_2_-adrenoceptors, there is no consistent relationship between intracellular cAMP and inhibition of Ca^2+^ signals under different stimulation conditions (Fig. 5C). This suggests an intracellular compartmentalization of the effective cAMP [28]. The cAMP produced immediately after activation of β_2_-adrenoceptors most effectively inhibits Ca^2+^ signals, but the sensitivity to isoproterenol is unchanged during sustained stimulation despite further accumulation of cAMP. This suggests that local regulation of histamine responses must continue throughout the sustained stimulation, but this is accompanied by diffusion of cAMP, which then accumulates in cytoplasmic regions where it does not effectively inhibit histamine responses (Fig. 5B). This slow accumulation of ‘ineffective’ cAMP would provide an explanation for our observation that as cAMP accumulates during sustained isoproterenol stimulation neither the maximal inhibition of histamine-evoked Ca^2+^ signals nor their sensitivity to isoproterenol increases (Fig. 5B). But why should globally distributed cAMP arising from inhibition of cyclic nucleotide PDEs appear more effective than cAMP delivered from β_2_-adrenoceptors (Fig. 5C)? We suggested previously that signaling from AC-coupled GPCRs to effector systems may occur within ‘hyperactive’ signaling junctions [43,54,55] (Fig. 7B). These, we propose, serve as digital switches, wherein activation of a junction generates more cAMP than required to fully activate associated PKA. The concentration-dependent effects of extracellular stimuli are proposed to arise from recruitment of active junctions, rather than from graded activity within individual junctions (Fig. 7B). Hence, each junction would behave as a robust on-off switch, locally saturating the neighboring PKA for as long as the GPCR stimulates AC. The benefits of this mode of signaling include speed, reliability and opportunities for local targeting of cAMP. Since inhibition of histamine-evoked Ca^2+^ signals by β_2_-adrenoceptors is associated with higher overall levels of intracellular cAMP than comparable inhibition with IBMX (Fig. 5C), we suggest that hyperactive cAMP signaling junctions mediate the communication between β_2_-adrenoceptors, PKA and histamine responses (Fig. 7B).

We conclude that cAMP selectively inhibits the Ca^2+^ signals evoked by histamine in hBASMCs. Communication between the GPCRs that stimulate AC and the PKA that mediates the inhibition occurs within hyperactive signaling junctions. These junctions, which may be a general feature of cAMP signaling, allow rapid, robust and specific communication between receptors and effectors [43,54,55].

## Author contributions

PD performed and analysed most experiments. VH completed the MS analysis of cAMP samples, with input from MRD. CWT supervised the project and contributed to analysis. CWT. CWT and PD wrote the paper. All authors reviewed the paper.

## Conflict of interest

VH and MRD are employees of Novartis, which manufactures drugs used to treat respiratory diseases. CWT and PD declare that they have no competing financial interests.
